# Energy Reserves, Information Need and a Pinch of Personality Determine Decision-Making on Route in Partially Migratory Blue Tits

**DOI:** 10.1371/journal.pone.0163213

**Published:** 2016-10-12

**Authors:** Anna L. K. Nilsson, Jan-Åke Nilsson, Claudia Mettke-Hofmann

**Affiliations:** 1 Centre for Ecological and Evolutionary Synthesis (CEES), Department of Biosciences, University of Oslo, Oslo, Norway; 2 Department of Biology, Unit of Evolutionary Ecology, Lund University, Lund, Sweden; 3 School of Natural Sciences & Psychology, Liverpool John Moores University, Liverpool, United Kingdom; Universita degli Studi di Milano-Bicocca, ITALY

## Abstract

In facultative partial migrants some individuals in a population are migratory and others are resident and individuals decide each year anew which strategy to choose. While the proportion of birds migrating is in part determined by environmental conditions and competitive abilities, the timing of individual departure and behaviours on route are little understood. Individuals encounter different environmental conditions when migrating earlier or later. Based on cost/ benefit considerations we tested whether behaviours on route were affected by time constraints, personality and/or age in a partially migrating population of Blue tits (*Cyanistes caeruleus*). We captured female Blue tits on migration at the Southern tip of Sweden during early, peak and late migration and measured latency to feed in an unfamiliar environment, exploration of a novel object and hesitation to feed beside a novel object (neophobia). Lean birds and birds with long wings started feeding earlier when released into the cage indicating that foraging decisions were mainly determined by energetic needs (lean and large birds). However, juveniles commenced feeding later with progression of the migratory season in concordance with predictions about personality effects. Furthermore, lean birds started to explore earlier than birds with larger fat reserves again indicating an effect of maintaining threshold energy reserves. Moreover, late migrating juveniles, started to explore earlier than early migrating juveniles possibly due to time constraints to find high-quality foraging patches or a suitable winter home. Finally, neophobia did not change over the migratory season indicating that this behaviour is not compromised by time constraints. The results overall indicate that decisions on route are mainly governed by energetic requirements and current needs to learn about the environment and only to a small extent by differences in personality.

## Introduction

Partial migration describes the phenomenon that some individuals in a population are migratory during the non-breeding season, whereas other individuals of the same population remain on their breeding ground [1]. It is a widespread phenomenon [2] occurring in fish [3], mammals [4] and birds [5–6]. Which individuals migrate in a population can be a) genetically determined, i.e. be fixed across lifetime, b) condition (age, sex, competition) or environmentally (resources, temperature, predation) dependent and therefore change within an individual’s life or c) be a mixture of both, genetic and condition/environment [7]. Recently, the latter has received increasing behavioural and genetic support [8–9].

While considerable research has been devoted to the causes why some individuals in a population migrate, there is little known about the proximate factors for an individual’s decision when to migrate, but see [10] and which factors govern decision-making on route. For example, in many partially migratory systems, environmental conditions such as limitation in resources leads to intense competition which causes subordinate individuals or less competitive ones (often young individuals and females) to migrate [11–14]. While median migration dates can vary with the magnitude of migration in a given year [15], the variation around this median in each year is little understood.

Timing of migration may have substantial effects on the conditions encountered during migration which are likely to influence decision-making on route. For example, early migrants may have less competition from other migrants at stopover sites, encounter good foraging conditions and have more time available to find a suitable winter home than late migrants who may be more time constrained to settle down. The latter is supported by faster migration speeds of late migrants [12, 16].

Migrants have to balance costs and benefits of different traits during migration [17]. While they have to collect information about their environment to find food and identify threats e.g. predation pressure, they spend only short periods of time at each site and should keep exploration to a minimum to save energy and time [18]. Late migrants may, therefore, invest less time in information gathering than early migrants and may possibly take greater risks.

The above scenario implies that the decision to migrate and behaviours on route are governed by the prevailing conditions such as being more time-constrained when late. In addition, recent research suggests that the decision to migrate may also be linked to an individuals’ personality [2]. Personality describes consistent behavioural and physiological differences between individuals with traits often correlated across situations [19–20]. In roach (*Rutilus rutilus*), migratory individuals were bolder (emergence from refuge into a novel environment) than resident ones [2] and timing of migration was consistent among individuals [21]. Furthermore, in humans personality affects movement tendencies. While these movements possibly resemble more dispersal, from a cognitive point of view they confront an individual with the same challenges as during migration. In both cases individuals have to decide whether to move and if so they are confronted with unfamiliar environments, threats and possibly varying resources. People open to experience and with low agreeableness were more likely to leave home, e.g. [22–23]. High neuroticism also predicted movement tendencies [24] and Jokela et al. found a dual role of high emotionality (experience of fear and anger) on movement tendencies [25]; while high emotionality predicted movement, it also predicted shorter movement distances. Likewise, dispersal tendencies in different taxa are often linked to a bold, less sociable and either high or low aggressive personality, reviewed in [26–27]. In birds and fish, dispersing individuals were more explorative than philopatric ones when tested before or after dispersal had taken place [28–29].

The idea that personality affects migratory decisions is exciting as it implies that individuals with specific behavioural characteristics are more likely to migrate than others. In partially migratory species with resource competition and dominance-linked migration patterns, individuals have to decide each year anew whether to migrate or not, and even birds with a resident personality may be forced to migrate, particularly when belonging to a subordinate category and cyclic food resources, e.g. beach mast are in low supply [15, 30].

Moreover, personality differences may not be distinct (resident or migratory) but gradual [31–32], resulting in a higher or lower behavioural and physiological propensity to migrate which may affect timing of migration. Such intermediate migratory phenotypes were suggested by Pulido in the Environmental Threshold Model of Partial Migration [8]. In this model migration is a quantitative genetic trait with intermediate individuals (between resident and migratory) being influenced by the environment. Movement patterns in partially migratory blackbirds (*Turdus merula*) support this model [14]. With respect to personality, traits may have evolved alongside migration tendencies. For example, individuals with a high migratory tendency may also have a suite of personality traits advantageous for migration (migratory personality) which prepares them for challenges on route such as dealing with unfamiliar environments, but they may be less adapted to deal with challenges on the breeding ground (e.g. aggression, competitiveness). As a consequence, those individuals may decide to leave early. In contrast, individuals with a low migratory tendency may have a suite of personality traits advantageous for residency (resident personality) such as high aggression and competitiveness and may try to stay on their breeding ground and only leave if they have to (as shown for blackbirds–[14]), for instance due to competition which may be higher later in the season as resources decline. They may be less adapted to deal with unfamiliar environments. Individuals with an intermediate migratory tendency may also be intermediate in their personality regarding a migratory or resident personality and may have migration departures timed between birds with a migratory or resident personality. Once on migration, birds with more or less migratory personalities may again differ in their decisions, i.e. how they vary in their response to challenges while on migration.

The Blue tit (*Cyanistes caeruleus*) is a partial migrant in some of its distributional range. Migration is largely driven by density and food availability (beech mast) during autumn [15]. High density and low food availability result in increased intraspecific competition and consequently sub-dominant categories, such as females and juveniles, are more prone to migrate [33–34]. In some years eruptive-like migration may occur [15, 33]. Behavioural traits have been found to differ between individuals adopting different migration strategies as migrating blue tits are more explorative than residents [35]. Together with a migration period of about one month [15], this offers the ideal opportunity to investigate whether a) specific behavioural traits differ along the migratory window (early-late migration) and whether any such differences can be explained with b) time-constraints or c) personality types.

We captured Blue tits of a partially migratory population at the Falsterbo Bird Observatory at the southern tip of Sweden during early, mid and late autumn migration in 2007 –a year with large numbers of blue tits migrating. Birds passing through this area originate mainly from Sweden and are characterised by very slow (median 13 km/day) and short distance (median 83 km) migration that is synchronised across large parts of the distributional range [12]. We restricted our study to females as both juveniles and adults participate in migration to a considerable amount, whereas adult males rarely migrate [15]. This allowed us to consider the effect of experience (age) on decision-making during migration as older individuals should have more experience in social interactions [36] and may also have migrated before [30], whereas juveniles are migratory naïve. We recorded a) the latency to feed when released into a novel environment as a measure of how quickly the bird adapts to the new environment; b) the latency to approach and touch a novel object in the familiar cage as a measure of boldness; and c) the duration of avoiding to feed when a novel object was placed beside the familiar food (neophobia). We formulated several hypotheses about how birds may respond in the experimental conditions over the migration period (early to late) based on cost-benefit considerations outlined above and what can be predicted from personality studies.

1.) Time-constraint hypotheses: With progression of the migratory season birds may become increasingly time-constrained to get away from unfavourable areas and/or find a winter quarter, which may have the following effects on behaviour.
  1.1.a) Early migrants are less time-constrained than late migrants and can afford to explore the environment more thoroughly (e.g. to assess habitat quality but also in terms of finding a suitable winter home) in accordance with slower migration speeds in early (September) than late (October) migrants of the same population [12].
  1.1.b) Alternatively, late migrants may explore more than early migrants as the former are severely time constrained *to find* a suitable area to settle down. Late migrants initially have a high migration speed but quickly slow down [12] which could be linked to more intense exploration.
Prediction 1.1.a is more concerned about the time pressure to move away as conditions get harsher with progression of the migratory season, whereas prediction 1.1.b is more concerned about the pressure to find a suitable winter site late in the migratory season. There may be late migrants on their early part of migration (and hence being fast, prediction 1.1.a) and other late migrants on their latter part of migration (increasingly searching for suitable winter sites, prediction 1.1.b).
  1.2.) Early migrants are more neophobic than late migrants as they can afford to be cautious to reduce risks due to having more time available [37].
  1.3.) Early migrants may start foraging in an unfamiliar environment later than late migrants as they can afford to spend more time to assess risks.
The three traits are expected to be independent of each other and not correlated within an individual.
2.) Personality-related hypotheses: As mentioned above personality traits may have evolved together with migratory tendencies. Individuals with a strong migratory tendency may have personality traits that are advantageous for migration (migratory personality), whereas individuals with a low migratory tendency may have personality traits favouring remaining on the breeding ground (resident personality).
  2.1.) Individuals with a more migratory personality may start migration earlier than individuals with a more resident personality as they are cognitively well adapted to deal with challenges on migration (see above). Based on this assumption, early migrants are predicted to be more explorative [2, 23], more neophobic [24–25], and to start foraging in a novel environment earlier (as they settle in faster [38]) than late migrants in concordance with personality traits found in individuals that move away rather than stay in their familiar environment.
These traits may be correlated within individuals forming a behavioural syndrome (migration or resident syndrome, respectively). Predictions under 1 (time constraints) and 2 (personality) are in part mutually exclusive.
3.) Age-related hypotheses: Young and old birds differ in their experience both in social interactions and thereby in dominance, and in migratory experience *per se* as older birds may have migrated before [30], whereas juveniles are migratory naïve. Social and non-social experiences will affect behaviours on route.
  3.1.) Juvenile birds are expected to explore more than older birds as they are sub-dominant, have to learn about resources in an unfamiliar environment due to their lower experience and in addition have to evaluate sites for winter settlement, whereas old birds may be able to chase the young birds away from food resources and may know where to go to winter from earlier migrations. Young individuals are also often found to be more explorative than older individuals [39–43].
  3.2.) Young birds are expected to be more neophobic than older birds as they have less experience to compare new situations with old ones in concordance with other studies finding age-related differences in neophobia [42, 44].
  3.3.) Young birds may start foraging in an unfamiliar environment earlier than older birds as they may be more flexible and accept new food and situations quicker than adult birds.
The three traits are not expected to be correlated within individuals. Predictions under 3 are not necessarily mutual exclusive to the other predictions as age might interact with personality or time-constraints.

## Material and Methods

### Study species and site

Twenty-four migratory female blue tits were captured for experiments during the standardized ringing scheme in 2007 at Falsterbo bird observatory [45] located at the south-western tip of Sweden (55° 23’N, 12° 49’E). Swedish blue tits have a south-westerly migratory direction and follow leading coastlines [12, 15]. Due to the location of the ringing station, birds captured at the ringing station are all on migration and about to cross the sea [15], apart from two breeding pairs of local blue tits that are ringed before the migrants start to arrive. Only females were selected for the experiment as this is the only sex with considerable migration in both juveniles and adults. All birds started to feed within an hour after capture and were released with a higher body mass than at capture. All experimental protocols comply with national legislation and this study was specifically approved by the Malmö/Lund Animal Care committee.

Eight birds each were taken from the bird observatory at three different times during the autumn migration period; a) during early migration (23rd Sep.), b) during peak migration (3rd and 4th Oct.), and c) during late migration (13th Oct). Four birds were juveniles (< 1 year) and four adults (> 1 year) at each time. Birds were captured between 9.30 AM and 4 PM and immediately ringed and aged [46]. Wing length, fat score (following [46]) and body mass were recorded and birds were then transferred to individual cages (0.45 x 0.30 x 0.48 cm) indoors. Cages consisted of two upper, outer perches and one lower middle perch, a water suspensor and a feeder at the front of the cage. Birds were given a mixture of mealworms, *Tenebrio sp*. and sunflower seeds. All birds were kept in the same room with natural light patterns. Birds could hear but not see each other. Overall, birds were tested five times and then released. All experiments were video-taped.

### Experimental procedure

Birds were tested in three different experiments between 9.00 to 11.00 AM, except for experiment one which started when the bird was released into the cage. Either two or six birds were tested at a time due to the arrangement of the cages and availability of only three cameras. (1) On the day of capture, latency to feed was recorded on release into the cage as an indication of how quickly individuals adapt to their new situation. (2) Either on day 4 or 5 after capture (half of the birds, each), the bird’s neophilia (attraction to novelty) was tested by placing a novel object (red or green pyramid; 5 x 5 x 3.5 cm) on one of the upper perches for 30 minutes and recording the latency to touch the object. The object was placed at a neutral location in the cage that the bird was free to approach or avoid. In this situation, the novel object elicits approach (neophilia) and avoidance (neophobia) but in case the bird approaches, neophilia is stronger and a good indicator for an individual’s interest in the object [47]. (3) Birds tested on day 4 on neophilia were tested on neophobia (avoidance of novelty) on day 5 and vice versa. A novel object (orange or white round cotton mop; 7 cm in diameter) was positioned beside the feeder for 60 minutes and the latency to feed was measured. Additionally, the latency to feed after the same disturbance (starting of the cameras), but without the novel object, was measured on two days (control latency) within three days of the neophobia experiment. In the neophobia experiment, the bird is in a conflict between the motivation to feed and the motivation to avoid the novel object. The difference in time between feeding with and without the novel object reflects neophobia [47–48]. Neophilia and neophobia represent two independent motivations [47, 49–50] and also belong to two different personality dimensions [51–52]. On day 9 and 10, all birds were re-tested on the neophobia and neophilia test using the colour that was not used in the previous set-up (balanced design) to test for consistency of behaviours over time. The same object but a different colour was used to keep objects as similar as possible but not identical to avoid habituation [53] and retain novelty [54].

### Statistics

Five dependent variables were extracted from the experiments; a) latency to feed on the capture day, b) latency to explore (neophilia) on the first trial and c) on the second trial, d) latency to feed beside the novel object (neophobia) on the first trial and e) on the second trial. Neophobia latencies were calculated from the average time to feed without the novel object subtracted from the latency to feed with the novel object reflecting the neophobic reaction. Latency to explore was taken as a proxy for intensity of exploration as these variables were negatively correlated in an earlier study on the same population [35] as was the case in Great tits (*Parus major*) [55]. Note that a few birds did not feed or explore within the set time limits for experiments, leading to truncation of our data.

For latency to feed on the capture day we used ANOVA with fat (0–5 with zero indicating no fat) and wing length as an indicator of size as continuous variables and age (younger or older than one year) and migratory season (early—peak—late migration) as factors. We also included the interaction terms age x wing length (see below) and age x migratory season to test for differences between age classes along the migratory window. No three-way interaction terms were included because of the small sample size. Non-significant terms were removed in a backward elimination process, where main terms were retained if they were included in significant interactions. Predictor variables were un-correlated, with the exception of adult birds having longer wings (r_p_ = 0.51, df = 22, P = 0.010) and a trend for lower fat scores (r_p_ = -0.40, df = 22, P = 0.054) than juvenile birds. Time of the day at capture did not affect latency to feed (r_p_ = 0.30, df = 22, P = 0.148). Wing length, indicating the general size of the birds, did not change over season in our sample (b = 0.2, t = 1.6, P = 0.14).

For neophobia and exploration latencies we fitted linear mixed effect models (LMM) to consider individual variation with the same independent variables as before. These models were fitted with REstricted Maximum Likelihood (REML). ID of the bird was included as a random factor. Non-significant terms were removed in a backward elimination process, where main terms were retained if they were included in significant interactions. Interactions were further explored by re-fitting the model and shifting the reference levels of the categorical variables.

Fat and wing length were used to indicate how much body reserves the birds had stored in preparation for migration and as an indicator of general body size, respectively. Latency to feed, exploration latencies and neophobia were square-root transformed to approach normality. Because the neophobia data contained negative values (minimum = -149.5), we added 150 to all neophobia measurements. All three variables were also analysed using model selection based on Akaike’s Information Criterion corrected for small sample sizes, AIC_c_ [56] (see Tables A-C in S1 File).

Furthermore, consistency of neophilia and neophobia reactions was tested by comparing the first and second trial using Pearson’s correlations. Finally, we tested for a correlation between latency to feed on the capture day, exploration and neophobia for possible behavioural syndromes by use of Pearson’s correlations. In all correlations, square-root transformed variables were used. All analyses were conducted in the program R version 3.2.2 [57], with add-on packages ‘nlme’ [58] for mixed effects models.

### Ethical note

All birds started to feed within an hour after capture and were released with a higher body mass than at capture. Experiments conformed to Swedish regulations and were conducted under permit no M237-07.

## Results

**Fig 1:**
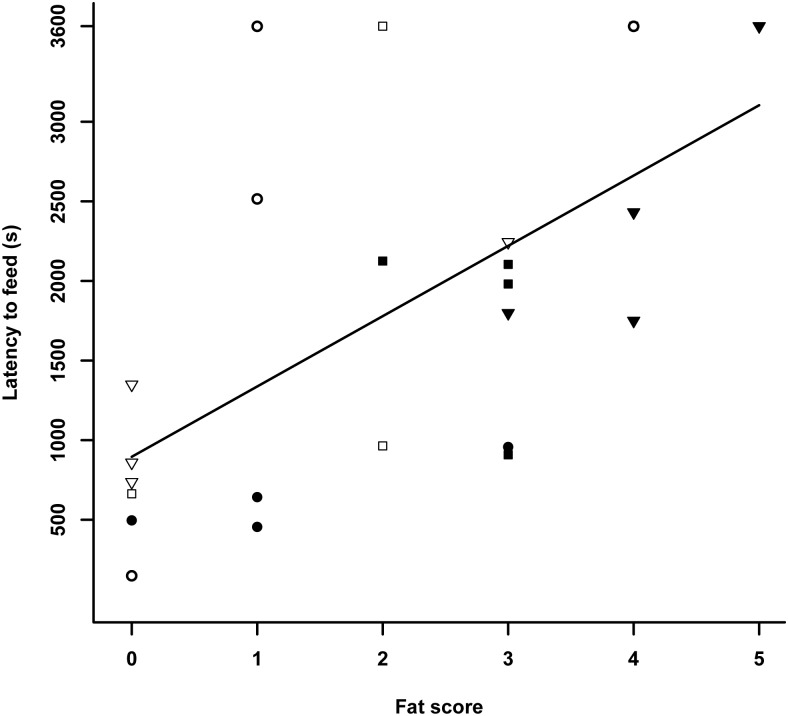
Latency to feed (s) after release in the cage on the day of capture in relation to fat scores (0 = no fat) with early (circles), mid (squares) and late migratory season (triangles) in juvenile (filled symbols) and adult (open symbols) female blue tits in Falsterbo in autumn 2007.

**Fig 2:**
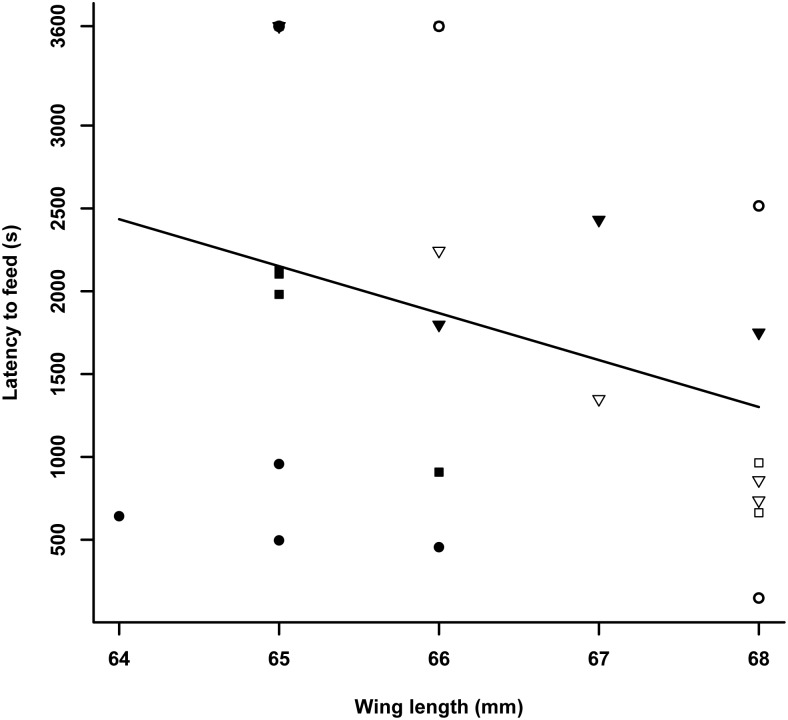
Latencies to feed (s) after release in the cage on the day of capture in relation to wing length (mm) with early (circles), mid (squares) and late migratory season (triangles) in juvenile (filled symbols) and adult (open symbols) female blue tits in Falsterbo in autumn 2007.

**Fig 3:**
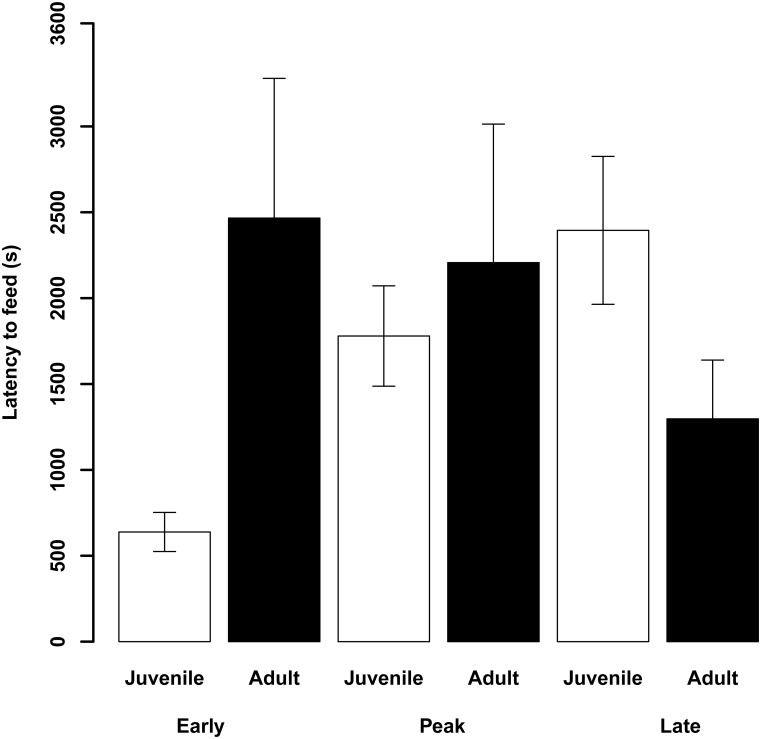
Mean (± SE) latencies to feed (s) after release in the cage on the day of capture during early, peak and late migration in juvenile (white bars) and adult (black bars) female blue tits in Falsterbo in autumn 2007.

Latency to feed on the capture day was significantly related to fat, wing length and the interaction between age and migratory season (Tables 1 and 2) explaining 79% of the variance. The higher the fat scores and the shorter the wing (i.e. the smaller the bird) the later the birds started foraging (Figs 1 and 2). Furthermore, when investigating the interaction between age and migratory season, we found that the feeding latencies of juveniles increased later in the migratory season, while there was no change among adults (ANOVA juveniles: F = 11.6, df = 2, P = 0.003; adults: F = 0.4, df = 2, P = 0.7; Fig 3). Model selection based on AIC_c_ (Table A in S1 File) favoured the model just based on fat, wing length and age without the interaction between age and migratory season.

**Table 1:** Latency to feed—Full model.

	Df	Sum Sq	Mean Sq	F value	P
Age	1	65.4	65.4	1.0	0.325
Fat	1	2680.1	2680.1	42.4	<0.0001
Wing length	1	440.3	440.3	7.0	0.019
Migratory season	2	71.9	35.9	0.6	0.578
Age x Wing length	1	115.8	115.8	1.8	0.196
Age x Migratory season	2	424.5	212.3	3.4	0.062
Residuals	15	947.9	63.2		

The full ANOVA model for latency to feed in the cage on the day of capture in female blue tits in Falsterbo autumn 2007

**Table 2:** Latency to feed—Restricted model.

	Df	Sum Sq	Mean Sq	F value	P
Age	1	65.4	65.4	1.04	0.324
Fat	1	2680.1	2680.1	42.5	<0.001
Wing length	1	440.3	440.3	7.0	0.018
Migratory season	2	71.9	35.9	0.6	0.578
Age x Migratory season	2	478.3	239.2	3.8	0.045
Residuals	16	1009.9	63.1		

The restricted ANOVA model for latency to feed in the cage on the day of capture in female blue tits in Falsterbo autumn 2007.

**Fig 4:**
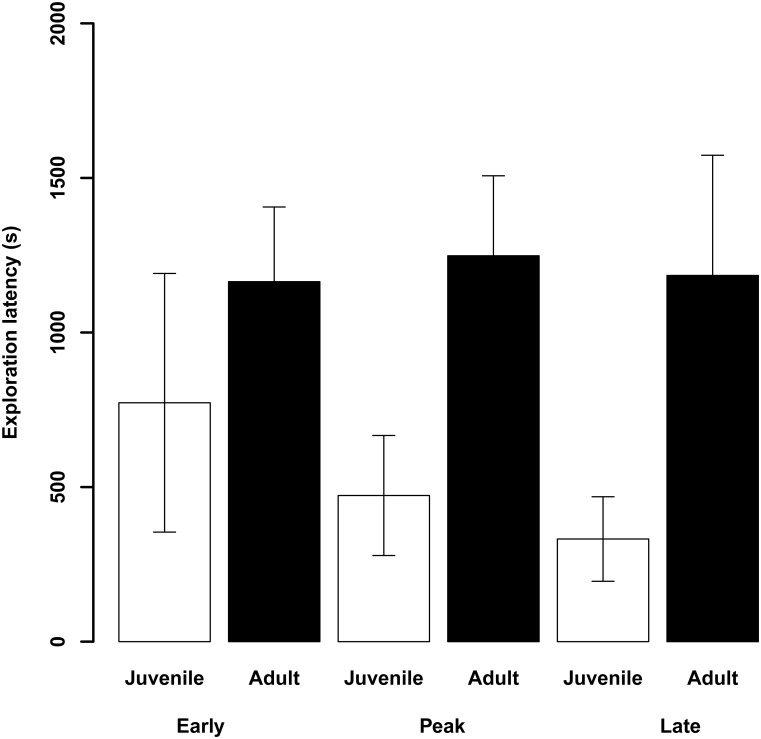
Mean (± SE) latencies (s) to touch the novel object (mean of the two exploration days) during early, peak and late migration in juvenile (white bars) and adult (black bars) female blue tits in Falsterbo autumn 2007.

In the exploration experiment, the restricted model with fat score, migratory season and the interaction between age and migratory season showed a significant influence on the latency to touch the novel object (Tables 3 and 4). Exploration latencies decreased with the advance of the migratory season (n = 23 as one novel object dropped to the ground). Furthermore, lean birds started exploring the novel object earlier than birds with high fat scores. Finally, while adult birds did not change exploration latency across the migratory season (early-peak: t = 0.1, P = 0.9; early-late: t = 0.3, P = 0.7; peak-late: t = 0.4, P = 0.7), juvenile birds decreased latency to explore with increasing migratory season (early-peak: t = -1.8, P = 0.09; early-late: t = -2.6, P = 0.02; peak-late; t = -1.2, P = 0.3; Fig 4). The random effect, individual, explained 22% of the variation in exploration latencies in the restricted model (individual: SD = 6.40; Residual: SD = 9.25). Model selection based on AIC_c_ revealed the same results (Table B in S1 File).

**Table 3:** Exploration latency—Full Linear Mixed effects Model.

	Df	Value	Std.E	T value	P
Intercept	23	-355.3	459.5	-0.77	0.447
Wing length	17	6.0	7.1	0.84	0.410
Fat	17	4.9	2.1	2.31	0.034
Age	17	201.0	289.5	0.69	0.497
Migratory season	17	-26.3	11.2	-2.35	0.031
Age x Migratory season	17	13.8	6.5	2.12	0.049
Age x wing length	17	-3.2	4.4	-0.72	0.481

Random factor: individual SD = 6.80, Residual SD = 9.27

Full Linear Mixed effects Model (LMM) results for latency to explore in female blue tits in Falsterbo autumn 2007.

**Table 4:** Exploration latency—Restricted Linear Mixed effects Model.

	Df	Value	Std.E	T value	P
Intercept	23	29.8	16.2	1.84	0.078
Fat	19	5.0	1.6	3.13	0.006
Age	19	-3.3	10.7	-0.30	0.764
Migratory season	19	-22.3	8.8	-2.52	0.021
Age x Migratory season	19	11.7	5.4	2.16	0.044

Random factor: individual SD = 6.40, Residual SD = 9.25

Restricted Linear Mixed effects Model (LMM) results for latency to explore in female blue tits in Falsterbo autumn 2007.

In the neophobia experiment, no relationship was found between neophobia latencies and any of the independent variables (Table 5). Individual variation explained about half of the variation in neophobia (full model, individual: SD = 10.51; Residual: SD = 9.74). Model selection based on AIC_c_ revealed the same results (Table C in S1 File).

**Table 5:** Neophobia latencies.

	Df	Value	Std.E	T value	P
Intercept	24	636.5	604.4	1.05	0.303
Wing length	17	-9.2	9.4	-0.99	0.337
Fat	17	2.8	2.7	1.04	0.311
Age	17	-397.1	372.5	-1.07	0.301
Migratory season	17	-1.3	14.5	-0.09	0.927
Age x Migratory season	17	1.1	8.4	0.13	0.896
Age x wing length	17	5.9	5.7	1.05	0.311

Random factor: Individual SD = 10.51, Residual SD = 9.74

The full Linear Mixed effects Model for neophobia latencies in female blue tits in Falsterbo autumn 2007.

Neophilia latencies in the first and second trial were positively correlated (r_p_ = 0.61, df = 21, P = 0.002). Because of the age effect on the first trial we tested for consistency within each age class. There was a non-significant positive trend in the same direction as in the full sample in both age classes (juveniles: r_p_ = 0.46, N = 12, P = 0.13; adults: r_p_ = 0.59, N = 11, P = 0.058). Also neophobia latencies of the first and second trial, showed a significant positive correlation (r_p_ = 0.49, df = 22, P = 0.015). Latency to feed, exploration and neophobia latencies were not correlated with each other (latency to feed-exploration r_p_ = 0.24, df = 21, P = 0.3; latency to feed-neophobia (first trial) r_p_ = 0.-02, df = 22, P = 0.9; exploration-neophobia (first trial) r_p_ = 0.13, df = 21, P = 0.6). All correlations are based on square-root transformed data.

## Discussion

Behavioural decisions during migration were primarily influenced by energetic conditions and current information needs and to a lesser degree by personality. Latency to feed after release into the cage was positively related to fat score and wing length indicating that energetic requirements were the main driving factors to start foraging. However, juveniles during early phases of migration started to feed sooner than during peak and late migration indicating some effects of personality (prediction 2.1) though this result was not supported by all models. Furthermore, latencies to explore a novel object were affected by fat scores (and hence energetic requirements) with lean birds exploring earlier than birds with higher fat scores. Birds also started exploration earlier the later they migrated in the season (supporting prediction 1.1.b) which was primarily driven by juvenile birds. The need to find a suitable winter site in combination with the lower dominance status of juveniles may explain this increase in exploration. Finally, neophobia was not affected by any of the studied variables. While exploration and neophobia latencies were consistent over time indicating personality traits [19, 59–60], none of the three investigated variables (including latency to feed) were correlated with each other, thus, the traits did not form a behavioural syndrome.

Due to the very brief migration period of the studied Blue tit population and logistic problems of testing more birds at a time we were not able to test more birds in each period. This resulted in relatively small samples sizes (n = 4) when interactions with age were included. Our non-significant results in those comparisons should therefore be interpreted with caution.

We used latency to feed on the capture day as a measure of how quickly individuals adapt to an unfamiliar environment. Primarily, energetic conditions determined when a bird started to forage with leaner and larger birds starting earlier than fatter and smaller birds. In other studies, factors such as perceived starvation or predation risk were found to determine latency to feed in an unfamiliar environment [61–62]. Therefore, birds with no or low fat reserves may have started to feed earlier to avoid starvation. Similarly, wing length is an indicator of size and as large birds have higher energy requirements, e.g. [63] they may need to start foraging earlier than smaller birds, again supporting hypotheses linked to energy-requirements [62]. An interaction, bordering statistical significance between age and season (Table 2), indicates that juveniles, unlike adults, started to forage later the more the migratory season progressed (Fig 3). However, it should be noted that this interaction term was not included when using model selection based on AIC_c_ and the results should be treated with caution. This result in part supports hypothesis 2.1. about the effects of personality on foraging decisions. The hypothesis was based on the assumption that birds with a more migratory personality would depart earlier as they are better adapted to deal with unfamiliar situations. This seems to be the case as only this hypothesis predicted the observed outcome. Birds with a more migratory personality may be at ease to leave the breeding ground due to their behavioural characteristics and may do so early to a) avoid suffering competition on the breeding ground and b) exploit favourable conditions on migration. Due to their migratory personality they may be less stressed and settle earlier than birds with a more resident personality as an adaptation to regularly encountering unfamiliar environments. This is in concordance with findings in the long-distance migratory garden warblers (*Sylvia borin*) that hesitated little to enter a novel environment as compared to closely related resident Sardinian warblers (*Sylvia melanocephala*) [38]. In another long-distance migrant, the sedge warbler (*Acrocephalus schoenobaenus*), latency to forage was part of a personality syndrome with birds commencing foraging early in a novel environment being more explorative, less nervous and better oriented in a cage [64]. However, in our study latency to feed was not correlated to any other variables. More research into this exciting effect of personality on decision when to migrate is needed.

Lean birds also explored novel objects earlier than birds with higher fat scores. Lean birds may approach and investigate unfamiliar structures earlier than fatter birds to identify new food resources. Similar results were found in reed warblers (*Acrocephalus scirpaceus*) when lured down during migration. Lean birds covered a larger exploratory distance than fat birds which did not move at all [65]. Likewise, lean sedge warblers explored an unfamiliar environment more than fat birds [64]. The results contradict studies modelling reactions to uncertainty that predict more exploration the higher the energy reserves [66–67] and studies on starlings (*Sturnus vulgaris*) that invest more time in information gathering the less hungry they are [68]. However, these studies did not address migratory conditions when different cost/benefit considerations may apply.

Birds also became more explorative with progression of the migratory season which was mainly driven by juveniles (Fig 4). This is in concordance with hypothesis 1.1.b which predicted that later migrating birds are forced to explore more to find a suitable winter site or high quality foraging patches to be able to move on. It also supports hypothesis 3.1. which predicted more exploration in juvenile birds. As young birds are sub-dominant to older ones, they need to be choosier when selecting stopover sites and wintering areas to avoid sites with high intra-specific competition. Long-term data on this Swedish population show that late migrants after an initial fast migration speed slow down migration [12]. The current data indicate that the slower migration speed may serve information gathering, particularly in juvenile birds. Adult birds may have migrated before but juvenile birds are naive in respect to their winter site and may continuously collect information about the environment for settlement decisions. This may be particularly important at the end of the migratory season when pressures to find a winter site increase. Being an extremely slow and short-distance migrant, juvenile blue tits may have a flexible response to their environment in order to find suitable winter habitats. In contrast, adult birds may have migrated already in earlier years and may know exactly where to go [12, 15] and do not have to invest much time in exploration or increase exploration later in the migratory season. Their exploration latencies therefore stayed the same throughout the migratory season and were consistently higher than in juveniles (Fig 4). Similar results were found in the obligate migratory Wilson’s warbler (*Wilsonia pusilla*) [69]. Juveniles showed longer exploratory movements during stopover than adults and movements increased over the migratory season. Paxton et al. interpreted these findings with the subordinate social status of the juveniles and their lower efficiency in finding resources [69]. Moreover, the seasonal effect was suggested to be linked to changes in resource distribution which may have required extended exploration to find adequate resources.

While clear neophobia reactions were shown that covered a broad range of latencies, neophobia did not vary with any of the measured variables and none of the hypotheses regarding time constraints, personality traits or age were confirmed. Neophobia protects an organism from encountering danger [70–72] and does not seem to be compromised even under the time constraints experienced at the end of the migratory season. Studies on Garden warblers (*Sylvia borin*) found that decisions to approach an unfamiliar object were more governed by consideration of risk (dangerousness of the object) rather than information gain [73] supporting the importance of neophobia in decision-making. However, other studies found that neophobia is plastic in response to e.g. predation risk [74–75]. In these latter studies, risk had actually changed, whereas in the warbler and the current study environmental uncertainty had changed or became more of an issue due to time constraints, respectively.

While exploration and neophobia were repeatable over time and therefore indicate the existence of personality traits [19, 59–60], they were not correlated with each other or with latency to feed. Furthermore, only one part of the personality-related hypotheses was confirmed. This may indicate that only this behaviour is linked to a migratory personality in this population. In earlier studies an effect of personality on decisions whether to migrate or not has been found in partially migratory study systems [2, 23–24] as well as in this population of blue tits where migratory individuals were more explorative than resident ones [35]. In those studies personality differences represented a clear cut between migrants and residents. Our study presents one of the first results showing a smooth transition from more to less migratory personality characteristics as the migratory season progressed which influenced decisions when to migrate and while on migration. This confirms our assumption that birds with a more migratory personality migrate earlier. Studies investigating timing of migration in birds and fish found that this trait is repeatable, i.e. individuals start migration at the same time each year [9, 76]. Whether this is the case in our Blue tit population needs further investigation.

To conclude, migratory decisions on route were primarily affected by energetic and current needs to gain information with a) lean birds starting foraging in an unfamiliar environment and exploration of a novel object earlier than birds with higher fat scores and b) earlier exploration in young birds later in the migratory season to speed up the process of finding a suitable winter home or high-quality foraging patches to refuel faster in order to carry on migration. While exploration and neophobia were consistent over time, personality traits had only a weak effect on behavioural decisions during migration.

## Supporting Information

S1 Data Supporting Data. (XLSX)

S1 File Model selection based on AIC_c_. (DOCX)
