# Supplementary material for: Energy Reserves, Information Need and a Pinch of Personality Determine Decision-Making on Route in Partially Migratory Blue Tits
Source: PLoS One. 2016 Oct 12;11(10):e0163213. doi: 10.1371/journal.pone.0163213 (PMC5061368; doi:10.1371/journal.pone.0163213)
Supplement: S1 File — (DOCX) [file pone.0163213.s002.docx]

Model selection based on AIC_c_

1. Latency to feed

Table A. Latency to feed in the cage on the day of capture in female blue tits in Falsterbo autumn 2007 (model selection based on AIC_c_ (AIC corrected for small sample size), Weights = Akaike weights). Model m1 (∆AIC_c_ = 11.6) corresponds to our full model and m2 (∆AIC_c_ = 7.1) to our restricted model in the analysis using backward stepwise elimination of variables.

| Model no | Model | AIC_c_ | ∆AIC_c_ | Weights |
| --- | --- | --- | --- | --- |
| m7 | Age + Fat + Wing length | 181.6 | 0.0 | 0.5498 |
| m5 | Age + Fat + Wing length + Age x Wing length | 183.2 | 1.6 | 0.2462 |
| m11 | Age + Fat | 184.4 | 2.7 | 0.1398 |
| m6 | Age + Fat + Wing length + Migratory season | 188.2 | 6.5 | 0.0210 |
| m2 | Age + Fat + Wing length + Migratory season + Age x Migratory season | 188.7 | 7.1 | 0.0159 |
| m18 | Fat | 189.4 | 7.8 | 0.0111 |
| m4 | Age + Fat + Wing length + Migratory season + Age x Wing length | 190.8 | 9.2 | 0.0056 |
| m8 | Age + Fat + Migratory season | 191.1 | 9.5 | 0.0048 |
| m14 | Fat + Wing length | 191.9 | 10.3 | 0.0032 |
| m1 | Age + Fat + Wing length + Migratory season + Age x Wing length + Age x Migratory season | 193.3 | 11.6 | 0.0016 |
| m15 | Fat + Migratory season | 195.4 | 13.8 | 0.0006 |
| m3 | Age + Fat + Migratory season + Age x Wing length + Age x Migratory season | 198.1 | 16.4 | 0.0001 |
| m10 | Fat + Wing length + Migratory season | 198.5 | 16.8 | 0.0001 |
| m12 | Age + Wing length | 199.5 | 17.9 | 0.0001 |
| m19 | Wing length | 199.6 | 18.0 | 0.0001 |
| m9 | Age + Wing length + Migratory season | 201.5 | 19.9 | 0.0000 |
| m17 | Age | 201.9 | 20.2 | 0.0000 |
| m16 | Wing length + Migratory season | 202.9 | 21.3 | 0.0000 |
| m20 | Migratory season | 203.7 | 22.1 | 0.0000 |
| m13 | Age + Migratory season | 206.6 | 24.9 | 0.0000 |

1. Latency to explore

Table B. Latency to explore in female blue tits in Falsterbo autumn 2007, modelled in Linear Mixed effects Models (LMM; model selection based on AIC_c_ (AIC corrected for small sample size), Weights = Akaike weights). Model m5 corresponds to the restricted model in the analysis using backward stepwise elimination of variables, where the full model corresponds to model m1.

| Model no | Model | AIC_c_ | ∆ AIC_c_ | Weights |
| --- | --- | --- | --- | --- |
| m5 | Fat + Age + Migratory season + Age x Migratory season | 369.6 | 0.0 | 0.3550 |
| m14 | Fat + Age | 372.0 | 2.4 | 0.1072 |
| m10 | Fat + Age + Migratory season | 372.1 | 2.5 | 0.1024 |
| m4 | Wing length + Fat + Age + Migratory season + Age x Migratory season | 372.1 | 2.5 | 0.1002 |
| m19 | Age | 372.8 | 3.2 | 0.0728 |
| m12 | Wing length + Age | 373.1 | 3.5 | 0.0622 |
| m7 | Wing length + Fat + Age | 373.1 | 3.5 | 0.0604 |
| m16 | Age + Migratory season | 374.4 | 4.9 | 0.0313 |
| m6 | Wing length + Fat + Age + Migratory season | 374.5 | 4.9 | 0.0312 |
| m1 | Wing length + Fat + Age + Migratory season + Age x Migratory season + Age x Wing length | 374.5 | 4.9 | 0.0300 |
| m9 | Wing length + Age + Migratory season | 375.5 | 5.9 | 0.0183 |
| m3 | Wing length + Fat + Age + Age x Wing length | 375.7 | 6.1 | 0.0167 |
| m2 | Wing length + Fat + Age + Migratory season + Age x Wing length | 377.1 | 7.5 | 0.0084 |
| m20 | Migratory season | 381.4 | 11.8 | 0.0010 |
| m17 | Wing length | 381.7 | 12.1 | 0.0008 |
| m18 | Fat | 381.8 | 12.2 | 0.0008 |
| m13 | Wing length + Migratory season | 383.4 | 13.8 | 0.0004 |
| m15 | Fat + Migratory season | 383.7 | 14.1 | 0.0003 |
| m11 | Wing length + Fat | 384.0 | 14.4 | 0.0003 |
| m8 | Wing length + Fat + Migratory season | 385.2 | 15.6 | 0.0001 |

1. Neophobia

Table C. Neophobia latencies in female blue tits in Falsterbo autumn 2007, modelled in Linear Mixed effects Models (LMM; model selection based on AIC_c_ (AIC corrected for small sample size), Weights = Akaike weights). From the Akaike weights, there was little support for any model apart from m21. The variables we measured are not likely to affect neophobia latencies, which is in concordance with the analysis using backward stepwise elimination of variables yielded.

| Model no | Model | AICc | ∆AICc | | Weights |
| --- | --- | --- | --- | --- | --- |
| m21 | 1 | 385.7 | | 0.0 | 0.2193 |
| m18 | Fat | 386.4 | | 0.7 | 0.1537 |
| m19 | Age | 386.7 | | 1.1 | 0.1289 |
| m17 | Wing length | 387.4 | | 1.7 | 0.0945 |
| m20 | Migratory season | 388.0 | | 2.3 | 0.0694 |
| m14 | Fat + Age | 388.4 | | 2.7 | 0.0568 |
| m11 | Wing length + Fat | 388.7 | | 3.0 | 0.0482 |
| m15 | Fat + Migratory season | 388.9 | | 3.2 | 0.0441 |
| m12 | Wing length + Age | 389.2 | | 3.5 | 0.0386 |
| m16 | Age + Migratory Season | 389.2 | | 3.5 | 0.0386 |
| m13 | Wing length + Migratory season | 389.5 | | 3.8 | 0.0323 |
| m7 | Wing length + Fat +Age | 391.0 | | 5.3 | 0.0154 |
| m10 | Fat +Age + Migratory season | 391.0 | | 5.3 | 0.0153 |
| m8 | Wing length + Fat + Migratory season | 391.3 | | 5.6 | 0.0132 |
| m9 | Wing length + Age + Migratory season | 391.6 | | 5.9 | 0.0115 |
| m3 | Wing length + Fat +Age + Age x Age x Wing length | 392.2 | | 6.5 | 0.0086 |
| m5 | Fat +Age + Migratory season + Age x Migratory season | 393.6 | | 7.9 | 0.0042 |
| m6 | Wing length + Fat +Age + Migratory season | 393.7 | | 8.0 | 0.0039 |
| m2 | Wing length + Fat +Age + Migratory season + Age x Wing length | 395.0 | | 9.3 | 0.0021 |
| m4 | Wing length + Fat +Age + Migratory season + Age x Migratory season | 396.5 | | 10.8 | 0.0010 |
| m1 | Wing length + Fat +Age + Migratory season + Age x Migratory season + Age x Wing length | 398.0 | | 12.4 | 0.0005 |
